# What do community health workers have to say about their work, and how can this inform improved programme design? A case study with CHWs within Kenya

**DOI:** 10.3402/gha.v8.27168

**Published:** 2015-05-22

**Authors:** Martin Oliver, Anne Geniets, Niall Winters, Isabella Rega, Simon M. Mbae

**Affiliations:** 1 London Knowledge Lab, Institute of Education, University of London, London, United Kingdom; 2 Department of Education, University of Oxford, Oxford, United Kingdom; 3 Media School, Bournemouth University, Dorset, United Kingdom; 4 Amref Health Africa, Nairobi, Kenya

**Keywords:** community health workers, medical supervision, clinical referral, close-to-community

## Abstract

**Background:**

Community health workers (CHWs) are used increasingly in the world to address shortages of health workers and the lack of a pervasive national health system. However, while their role is often described at a policy level, it is not clear how these ideals are instantiated in practice, how best to support this work, or how the work is interpreted by local actors. CHWs are often spoken about or spoken for, but there is little evidence of CHWs’ own characterisation of their practice, which raises questions for global health advocates regarding power and participation in CHW programmes. This paper addresses this issue.

**Design:**

A case study approach was undertaken in a series of four steps. Firstly, groups of CHWs from two communities met and reported what their daily work consisted of. Secondly, individual CHWs were interviewed so that they could provide fuller, more detailed accounts of their work and experiences; in addition, community health extension workers and community health committee members were interviewed, to provide alternative perspectives. Thirdly, notes and observations were taken in community meetings and monthly meetings. The data were then analysed thematically, creating an account of how CHWs describe their own work, and the tensions and challenges that they face.

**Results:**

The thematic analysis of the interview data explored the structure of CHW's work, in terms of the frequency and range of visits, activities undertaken during visits (monitoring, referral, etc.) and the wider context of their work (links to the community and health service, limited training, coordination and mutual support through action and discussion days, etc.), and provided an opportunity for CHWs to explain their motivations, concerns and how they understood their role. The importance of these findings as a contribution to the field is evidenced by the depth and detail of their descriptive power. One important aspect of this is that CHWs’ accounts of both successes and challenges involved material elements: leaky tins and dishracks evidenced successful health interventions, whilst bicycles, empty first aid kits and recruiting stretcher bearers evidenced the difficulties of resourcing and geography they are required to overcome.

**Conclusion:**

The way that these CHWs described their work was as healthcare generalists, working to serve their community and to integrate it with the official health system. Their work involves referrals, monitoring, reporting and educational interactions. Whilst they face problems with resources and training, their accounts show that they respond to this in creative ways, working within established systems of community power and formal authority to achieve their goals, rather than falling into a ‘deficit’ position that requires remedial external intervention. Their work is widely appreciated, although some households do resist their interventions, and figures of authority sometimes question their manner and expertise. The material challenges that they face have both practical and community aspects, since coping with scarcity brings community members together. The implication of this is that programmes co-designed with CHWs will be easier to implement because of their relevance to their practices and experiences, whereas those that assume a deficit model or seek to use CHWs as an instrument to implement external priorities are likely to disrupt their work.

Community health workers (CHWs) represent a widespread strategy in the majority world to address shortages of health workers and the lack of a pervasive national health system, particularly in rural areas. Recently, research interest in this group has grown, both because of the need to accomplish the health-related Millennium Development Goals combined with the lack of human resources available for health work in the majority of the world. Community engagement in health systems has been seen as both a practical response to the challenging conditions of health provision in low-income settings [e.g. (1, 2)] and a key principle for strengthening health systems more generally (3). However, a substantive account of CHW's own view of their own practice has been lacking up until now, hindering the integration or alignment of CHWs to the formal health care system at the community level. It has been argued, for example, ‘that CHWs have privileged insights into the social determinants of health in communities […] there is a need for these insights to inform policy for both health and inter-sectoral policies and priorities. […] However, there is limited knowledge on the extent to which CHWs are given opportunities to feed into health systems priority setting and bring their embedded knowledge to health systems debates’ (4, p. 9). This paper addresses this gap by advocating for the ‘voice’ of CHWs to be a central component of programme design and implementation.

## Framing the role of CHWs

The concept of CHWs was universally adopted at the Alma Ata conference in 1978, as a means for achieving the goal of health for all. Although CHWs operate under a variety of names, there is evidence that the role has existed in one form or another for more than 50 years (5). However, this variety of terminology signals inconsistencies in what CHWs do, as well as how they are identified. Reflecting this, many definitions of their role have remained vague. For example, in 1989, The World Health Organization proposed that:

Community Health Workers should be members of the communities where they work, should be selected by communities, should be answerable to the communities for their activities, should be supported by health system but not necessarily a part of its organisation, and have shorter training than professional workers. (6, p. 4)

Whilst this outlined relationships, it did not specify in any detail what CHWs might be expected to know or do. To address this, Lehmann & Sanders (5) undertook an extensive systematic review of previous literature, and distinguished two kinds of CHWs: generalists and specialists. Generalists performed a wide range of activities, from preventive care to first aid, covering common health and social issues in the area, such as child and maternal care, HIV/AIDS, nutrition and environmental sanitation. Examples of this include Kenyan CHWs who promote behaviour change through health education, earlier case identification and timely referral to trained health care providers (1); and the ASHAs in India, who identify and register new pregnancies, births and deaths; mobilise, counsel and support the community in demanding entitled health services; identify, manage, or refer diseased cases; support health service delivery through home visits, first aid, immunisation sessions and camps; maintain data; and participate in community-level health planning (7).

The community-facing role of CHWs means that these duties are typically carried out within households, rather than in formal medical settings. Importantly, however, generalists also act as a link between community households and the nearest health facility. They may also organise community development activities and collect data from the households (8, 4).

The roles of specialist CHWs are different, focusing primarily on specific health issues of concern to the program they are enrolled in – such as maternal and child health, TB care, malaria control, or HIV/AIDS care (5). Lehmann & Sanders go on to note that it is ‘impossible to comprehensively summarize or even represent the range of activities of specialist health workers’ (p. 12), but do identify a range of exemplars, covering management, dispensing, surveillance and referral.

## The effectiveness of CHWs

The importance of such work in areas beyond the reach of national healthcare systems is well evidenced, but concerns remains that CHWs’ work is less effective than it could be: ‘there is no longer any question of whether CHWs can be key agents in improving health: the question is how their potential can be realized’ (6, p. 2). Kahssay et al. propose supervision, training, team-based approaches and support from community health committees (CHCs) as possible ways to improve CHWs’ practice. Interestingly, these are primarily deficit-based interventions, assuming that CHWs themselves are the issue, although influences such as the attitudes of health personnel or community members may also be problems. Moreover, such interventions have not resolved matters; although training and supervision have been recognised as being important for over 20 years (9), there remains considerable variation in what is available. For example, some programmes provide either no in-service training or only informal support, whereas others provide continuous training and/or refresher courses (10), and Hill et al. (11) identified that supervision only contributes to the quality of CHW's work when it is of high quality and supportive, whereas the quality of current supervision varies considerably.

## The agency of CHWs

This body of prior research provides a useful high-level overview of CHWs practices. It outlines broad areas of responsibility, for example. However, there is relatively little ‘on-the-ground’ evidence about the role and practices of CHWs. The primary focus is on policy imperatives (what CHWs ‘ought’ to do) or special initiatives, which are not necessarily representative of regular, day-to-day practice, and which assume CHWs operate as an instrument of policy rather than as gatekeepers or negotiators who moderate and reinterpret initiatives to ensure their viability or relevance. Braun et al. (12), for example, undertook a systematic review of the use of mobile technologies to support CHWs. Whilst this review identified common practices such as home visits, assessment and treatment of disease, data collection, education and counselling and referrals, it did at a high level of generality, signalling categories of work but providing few details about the processes involved. Moreover, the projects reviewed were exceptional, typically undertaken through funded initiatives, with no guarantee of the sustainability of these new practices.

Much of the research to date reports on what CHWs do through such high-level categorisations; it does not reflect, for example, how CHWs themselves see their work. This is a particular issue for those wishing to understand CHW's work, since there is little evidence of participatory approaches, through which the CHWs would be able to explain what they do. This is an issue: CHWs and other close-to-community (CTC) providers may originally have been introduced as a means of extending services, but in their operation have developed insights and expertise into the operation of initiatives that it not currently taken into account in policy or new initiatives (4).

Qualitative studies have been undertaken to explore and understand implementation issues. For example, Mireku et al.'s interviews with service users, service providers (including CHWs) and with health managers and national policy makers (13) identified that CHWs were well accepted, but confirmed the challenge of regular visits, geographical coverage and the availability of services, transport and supplies. They also found that their workload was not clearly defined; that supervisors often prioritised facility-based responsibilities over community work and had no clear guidelines for supervision; that there are few incentives (beyond community recognition) for their work; and that CHWs were sometimes forced to use their own resources to subsidise services.

Other studies have also identified some insights from CHWs, or from those who work closely with them. For example, Nyamhanga et al. (focusing on community-level disease prevention and health promotion) included accounts from ward-level or village-level officers and committee members, revealing day-to-day challenges to implementation such as the underfunding of education and intervention activities, lack of knowledge and skills for planning and supervising community health programmes, and limited community participation (14). Similarly, Buchner et al. interviewed stakeholders (not CHWs directly), revealing the importance of a sense of trust in achieving health and economic benefits (15). Pitt et al.'s interviews with CHWs in relation to malaria prevention also identified misinformation and suspicion as issues for implementation, and also revealing how the distribution of scarce resources was understood as a ‘gift’, creating a sense of obligation within the community (16).

Although these studies recognising some of the expertise of CHWs, this is a secondary function, a by-product of the main purpose of the work. What CHWs have to say about their own practice remains largely unheard. Although there is over two decades of literature addressing CHWs, much of this is policy-driven, taking a directive, normative tone. Other research involves CHWs as part of the health system, exploring for example their efficacy in delivering specific interventions. Accounts that explore the day-to-day realities of working as a CHW, and how the CHWs themselves understand and explain these, are rarer. Such accounts remain relatively fragmented, providing little detail about how their work is actually undertaken, or about how the perspective of their communities on their work. This is a missed opportunity, given the expertise that CHWs have about building links between formal healthcare and the community, and about the successful implementation of new initiatives.

This paper seeks to address this gap by presenting the findings of a study that explored the day-to-day practices of CHWs in two regions of Kenya (although we believe that similar challenges arise in other contexts), through the generation of rich qualitative data with CHWs and key members of the communities they serve. In doing so, it contributes to previous literature both by detailing what practice consists of in these regions, and by exploring what this work means to the CHWs.

## Methodology

The findings reported in this paper are based on a structured, in-depth qualitative case study of two communities in Kenya – one urban and one rural. The study was part of a project exploring the development, implementation and evaluation of a practice-based mobile learning intervention. The aim of the study reported here was to develop a thorough and systematic account of CHW's practices, providing a baseline to inform the development and evaluation of subsequent interventions. Given the gaps identified in the literature above, particular concerns included the patterns of communal engagement and support, and the training and supervision of CHWs.

A case study was chosen as the methodological approach because these are well suited to developing an in-depth understanding of contexts and issues, allowing for the ‘social construction of meaning in-situ’ (17, p. 33). The emphasis here is on the identification and description of issues as identified and understood by the CHWs and other CTC actors, as a precursor to theory development, claims about prevalence or other generalisations. Such cases can also contribute to theory refinement by generating interpretations, which can be useful in limiting other generalisations or identifying areas of complexity that warrant further study (18). Used in such ways, case studies support better informed understandings of factors influencing complex interventions (19), thus minimising the implementation variation (20) across sites when more generalisable methodologies (such as randomised control trials) need to be used. The kinds of analytical generalisations that case studies support also.

The study therefore explored CHWs’ roles and practices, generating an explanation of the day-to-day practices of CHWs in their own words, and allowing an investigation of how CHWs understand their roles. Specifically, the study invited them to reflect on the challenges they face, and the forms of support that might help them with these. A case study approach was required to explore these open-ended, practitioner-focused questions.

### Study setting

#### The locations

Both sites are located within Kenya. National statistics (21) show that the country is characterised by sharp contrasts between urban and rural households, for example in terms of access to improved water sources (90% urban, slightly over 50% rural) or electricity (around 65% urban, 8% rural), although access to mobile phones is comparatively high (86% of urban households, 53% of rural). Infant mortality nationally stands at 52 deaths per 1,000 live births, although mortality rates differ considerably by province. Home births are more common in rural than urban areas (63% compared to 25%) and only 44% of births are assisted by a doctor, nurse or midwife.

Amref's own internal statistics provide a more detailed picture, and are used here to help characterise the study sites. The first study site is a semi-arid rural county in Eastern Kenya which experiences long droughts, resulting in high poverty levels. The county has a low number of skilled health workers, low access for CHWs to continuing education to improve their capacity in service delivery, an estimated 86% shortage of CHWs and a doctor to population ratio of approximately 1:119,879. Health care services are delivered through an estimated 138 health facilities. Only 28% of women are recorded to deliver at health facilities (the national average is 46%), and only 42% seek four antenatal care visits, with 36% attending postnatal care services.

The second site is an urban informal settlement in Nairobi. Health care provision is extremely limited, poorly resourced and difficult to access, making the extended reach of CHWs critically important. This site was important because over 34% of Kenyans live in urban areas; 71% of these live in informal settlements. The community is characterised by high levels of poverty, insecurity and inadequate access to basic social services. There is little or no access to water, electricity, basic services and adequate sanitation. Most structures are let on a room-by-room basis with many families (on average six people, compared to a national average household of 4.2) living in just one room. These factors have serious health repercussions, demonstrated by the child mortality rate: for every 1,000 children born in Nairobi's informal settlements, 151 will die before the age of five (the average for Nairobi as a whole is 62).

While these two study sites cannot be treated as statistically representative of all instances of CHWs’ working contexts, they provide compelling evidence from two contrasting situations from which lessons can be learned.

### The community strategy and the role of CTC providers

Wide regional disparities in health services and shortages of human resources in the health sector make the availability and accessibility of health services in Kenya challenging. Prompted by these challenges in general and in response to deteriorating maternal and infant mortality rates specifically, in 2006, the Ministry of Health decided to decentralise the provision of health services and to devise a new health strategy, the *Kenyan Community Health Strategy* (22). A plan for the training and involvement of CTC providers on a regional level was designed and implemented in 2008. The administrative structure of this new community health strategy was divided into six levels: Level 6 – the national level; Level 5 – the provincial level; Level 4 – the district level; Levels 3 and 2 – the Health Facility Level; and Level 1 – the CHC level. This administrative and managerial decentralisation of the country's health service provision allowed the communities to participate in health decision making on levels 1, 2 and 3, i.e. at a community and at a health facility level. According to these administrative levels, a district health management team now manages the committee of the health facilities, who in turn manages CHCs, and the CHCs manage their voluntary CHWs. (CHWs in Kenya have been re-titled as Community Health Volunteers, or CHVs; however, the more widely accepted term, CHW, will be used throughout this paper.)

These voluntary CHWs are linked to primary health facilities through trained health workers employed in the facilities – called community health extension workers (CHEWs) (22). The CHEW's role followed the supervisory model outlined by Mireku et al. (13). Currently, two government-employed CHEWs supervise 50 voluntary CHWs – although under revisions to the community health strategy it is proposed that in the future, five CHEWs will supervise 10 voluntary CHWs.

The voluntary CHWs are managed by their CHCs. The purpose of the CHC is to represent issues affecting the provision of health services in the communities and direct resources and CHWs towards them. According to the community health strategy implementation guide (22), the CHC's roles include:

- identifying community health priorities;
- planning community health actions;
- participating in community health actions;
- monitoring and reporting on planned health actions;
- mobilising resources for health actions;
- coordinating CHW activities;
- organising and implementing community health days;
- reporting to level 2 on priority diseases and other health conditions;
- leading community outreach and campaign initiatives; and
- advocating for good health in the community.

Members of the CHC are elected at the assistant chief's *baraza* (a meeting with community elders) and generally are elders or of respectable social status. Together, these administrative and managerial structures constitute the community health strategy and shape the roles of CTC providers.

### The role of Amref Health Africa in supporting CTC providers

The voluntary CHWs receive basic, 2-week long community health training from Amref Health Africa, Africa's largest International Health NGO (23). Amref Health Africa is an African-based organisation that aims to strengthen the capacity and capability of health and health-related professionals and institutions in Africa through training, research, health care provision and advocacy. Its mission is to ‘improve the health of people in Africa by partnering with and empowering communities, and strengthening health systems’ (www.amref.org/about-us/who-we-are/#sthash.7xI5xIoB.dpuf, accessed 1 March 2015).

Through a variety of different projects and partnerships, including e-learning initiatives and tailored community health courses, Amref Health Africa is training health workers in close to 35 African countries. It relies on an extended network of relations with governments, international donors and the private sector. In a 2010 report, Amref described itself as follows:

From its decades of engagement with Africa's most remote and impoverished populations, Amref has developed a specialised approach to its work in health. Much of its credibility with local communities and African governments stems from the relationship and trust that Amref has built over the past 54 years. Amref learns from, influences and partners with communities and community organisations; local and national governments and ministries of health; national and international NGOs and networks; global, regional institutions and donors to build long-term relationships and to ensure solutions are holistic and address the breadth of the communities’ health needs. (23, p. 4)

The study reported here was conducted with CTC providers working directly under the community health strategy, i.e. the voluntary CHWs, the CHEWs and the CHCs, in two communities in Kenya - one a rural community, the other an informal settlement.

### Study design

To address the gap in understanding CHW's practice identified above, a rich body of data was gathered through a series of four steps. Firstly, focus groups were held with groups of CHWs at which they were invited to talk through what they tended to do each day; these accounts were treated as data in their own right, but were also used to prompt mind-mapping activities that generated overviews of CHW practice. Following on from this, CHWs were given disposable cameras and asked to take images of their work, and the places in which this took place. This helped to ground the accounts of practice that CHWs gave. Thirdly, notes and observations of community meetings and monthly meetings contributed to a better understanding of the relationships between the CHWs as well as their supervisors, and the training needs and challenges identified by them. Finally, a series of interviews was undertaken with CHWs and representatives of the groups that were responsible for shaping their practice. The analysis presented in this paper focuses on the interview transcripts.

### Study population and sampling criteria

The population for this study was the individuals identified as CHWs by Amref, drawn from two of the communities they support. In addition, individuals working with the CHWs were invited to participate, to provide a wider context for their accounts.

The sampling was purposive, inviting participation to ensure a diversity in terms of region (rural community, informal settlement), gender, length of time in post and role (CHW, CHEW and CHCs). According to Mireku et al. (13, p. 22), ‘The CHC is the health governance structure adjoining the community; members are elected at the assistant chief's *baraza* (administrative meeting with community elders) to allow for representation of all villages in the CU.’ Their inclusion provided a community-led account of CHW's activities to be added to the analysis.

In all, the sample consisted of eight CHWs, four CHEWs and two CHC members).

### Data collection methods

The analysis in this paper draws on the interviews conducted with CHWs, CHEWs and CHC members (CHCs). A semi-structured approach was adopted, using themes identified through analysis of the preceding focus groups and visual mapping work. The interviews were conducted by two researchers, one from the UK partner and one from Amref; the UK researcher had spent time working with Amref *in situ* in order to develop sensitivity to the local culture and issues prior to beginning the interviews.

Sections of the interview were dropped where these were not relevant to the interviewee. This provided consistency across participants whilst remaining flexible enough to respect the time and interests of those being interviewed.

### Data processing and analysis

The interviews were transcribed by the research team. Each interview was analysed thematically. Transcripts were reviewed and exhaustively coded following a systematic process (24), and anonymised by replacing names with a tag, including reference to the field site (M – the rural site, or K – the informal settlement), interviewee's role (CHW, CHEW, CHC) and when multiple individuals from a specific site and role, a numeral (1, 2, etc.) to distinguish them. For example, MCHW1 refers to the first CHW interviewed at field site M.

Coding was crossed checked by two researchers, leading to refinement of the coding method. The coded extracts were then grouped and summarised. The relationship between code groups was considered, in some cases leading to the amalgamation of separate categories under superordinate groups. An important consideration was the relationship between routine and exceptional incidents: it would have been possible either to describe normal practice first, and then exceptions to it; or else to describe both normal and exceptional incidents of each kind of practice. The final decision was the latter, giving an overview of CHW's practices, and within each, describing both normal and exceptional incidents.

The resulting coding structure is reflected in the findings, below.

### Trustworthiness

In qualitative research, trustworthiness can be established by using well-established research methods, developing an early familiarity with the culture of participating organisations, triangulation, iterative questioning that provides depth and uncovers inconsistencies in accounts, frequent debriefing with the lead researchers and representatives of Amref through discussion of issues arising, peer scrutiny, ‘thick description’ of the case (25), and maintaining a detailed audit trail (26).

The steps outlined above provided early familiarity with the communities and the CHWs; triangulation was achieved through comparing the accounts provided by CHWs, including contrasts across the two field sites, and through the interviews with CHEWs and CHCs, which provided independent (and sometimes challenging) perspectives on CHW's work; the semi-structured interviews included sustained questioning about specific incidents in practice; the researchers engaged in regular debriefing during both data collection and analysis; and peer scrutiny and ‘thick description’ is provided in the reporting below. For the audit trail, field notes were taken during the empirical phase; coding notes were preserved; and coding decisions were recorded. For example, the quotes included below are all tagged, so that they can be traced back to specific interviews for context. Themes were identified independently, and consensus established through discussion, focusing on specific excerpts.

### Ethical considerations

The study followed the ethical guidelines of the British Educational Research Association and of Amref Health Africa, both of which required informed consent, guarantees of confidentiality and anonymity for participants, and the right of participants to withdraw and have their data removed. Care was taken to ensure that all participants understood that they were acting as volunteers, and were under no obligation to participate in the project. To avoid the influence of power relations on disclosure, the interviewers held no structural position in relation to interviewees, and in addition, the recordings and transcripts were kept confidential and anonymised, so that those with authority over participants had no access to the data set.

The ethical protocol, including briefing sheets and informed consent forms, received approval from the lead institution's ethical review board and from Amref.

## Findings

### CHW's areas of practice

#### Household visits

The core of CHW's routine work consists of visits to the households that have been assigned to them. Some CHWs visited their households weekly, others monthly. This seemed to depend on the case load that they had been assigned. The visits typically took 45 minutes to an hour. Some participants preferred to do their visits in the morning, so that data generated from the visit could be documented in the afternoon; one, however, undertook visits in the evening, because members of the family would be back from work at that point.

I usually wake up very early in the morning and I take breakfast. I have some cattle so I get food for them. Whenever am ready in my household I usually have a book, a pen and that log book in order to go and visit the household. That book is for report and those papers are for referring. (MCHW4)

These visits create the site for other community-facing practices, such as referrals, data collection, education and so on. Sometimes, CHWs are accompanied by a CHEW on their visits.

#### Medical intervention

A common expectation amongst clients was that CHWs would undertake some medical interventions.

There were cases where the CHW felt able to provide some first aid (in relation to minor burns, wounds and diarrhoea), but for the most part CHWs reported that this was not part of their role.

We are trained not to medicate, our job is to refer. (KCHW2)

Maybe as a CHW you can give first aid and it works but there are complicated cases that you see that because we CHW are not nurses or doctors, […] for example when you go to the household you find a child or a kid has high fever, that one we don't deal with. (KCHW3)

One important reason for this is that CHWs reported not having the resources they would need to make such interventions, even though clients expected them to.

They expect me to have first aid kit fully with all the items because they call us small doctors and nurses. So they expect us to have a fully equipped first aid kit and other services. (KCHW1)

#### Referrals

Part of the CHW's job is to ensure that anyone who needs the support of a health facility is sent there. Sometimes, such referrals arise in relation to crises or other exceptional circumstances; examples will be described later. Often, however, referrals are made on the basis of routine visits.

The relationship between me and the facility is that I bring patients from the households to the facility. If there is an expectant mother or a mother who has had a home delivery I send them to the facility as quickly as possible and if it is a sick person I refer. We have a very good relationship which must be there. I am a link between the community and facility. (MCHW2)

The CHWs reported that referrals happened ‘whenever I find a sick person or a child who needs immunisation’ or when someone was pregnant. If the CHW was able to deal with the situation they did so; but where the situation was unclear, required medicine or some other intervention, the client was referred to a health facility. Referral happened, therefore, when a CHW was unsure about the diagnosis, as well as when they were sure that it was beyond them to resolve; at times, CHWs were instructed to refer certain symptoms automatically (e.g. diarrhoea as a possible symptom of cholera).

I went to a household and I noticed there was a man who was coughing then I asked him for how long he had coughed. He told me two weeks. He was very ill. He had lost his appetite then I thought maybe it's not just a flu so I referred him to hospital for test or further investigation. (KCHW3)

Practice varied as to how, if at all, the health facility was informed about the referral case. The interviewees explained that they were expected to complete a Ministry of Health form for each referral; however, several CHWs said that because the forms had not been provided, ‘we write the name of the person, age, residence and what he/she is suffering from’ (MCHW4), and then the person is sent with the note to present themselves at the facility. Sometimes the CHW called the facility using a mobile phone, particularly if they wanted to discuss the circumstances for the referral; at other times the CHW accompanied the person, or gathered other people to help transport them (by motorbike, or, if necessary, by asking relatives or neighbours to help carry them on a stretcher). Accompanying them was particularly important if the person was unable to make the journey on their own, or else was unwilling to do so. Such arrangements relied on community support and raised questions about resourcing the work.

If you were refer four patients to [the district hospital] in a day you will be using a lot of money. If you were to use a stretcher to [the district hospital] then you need a large work force. That large work force would be dedicated to doing the work of the community. So what are they going to do at the end of the day, they are going to go back to their homes they don't have something to eat. And then again because of the problems we have here it would really be good if we had let's say an ambulance. (KCHEW1)

In other cases, the CHW was simply able to wait for the person to visit the facility by themselves, and return. If the CHW has left by this point, they may make a follow-up visit after a few days.

There were also variations over how to check that the client had actually attended the facility. Ideally, the Ministry form would be used to document the referral and would be sent back with the client to the CHW.

Once the person/client who has been referred reaches the health facility, the person receiving or the person who is going to attend that is the health care worker who is going to attend to the client is supposed to counter sign and will take that form, the countersigned one to the referring CHW so that at the end of the day the CHW is able to account that the client they had referred has reached the health facility. (KCHEW1)

However, since the forms were not always used, the CHW sometimes needed to contact the facility directly to check on the client.

The only way to know whether the client was referred is by following up at the reception ask whether the client, because you have the information, has come. The reception will give you the information and you are very accurate that the client came this day, was visited this way and was attended this way; so the work is smooth. (KCHW1)

As well as serving an important health function, referral also enabled the CHW's practice to be monitored. In part, this is visible simply from the volume of work facilities experience.

We have had a great milestone as far as the number of deliveries that we are conducting in the facility and the number of clients we see on a daily basis. We have seen it increase since we dispatched the CHWs to the ground. We were having 2/3/4 deliveries in a month but now we are having an average of 20 deliveries in a month, which is a great achievement. An average of 40 patients in a day in such a small facility from 10 in a day, it's a great thing. They are having good impact. You can see the patients are proud handing you that referral letter. It's really changing the whole area. (MCHEW2)

Again, this is simplified where the Ministry forms are available.

Referral system also gives the accuracy of the job; how effective you are to the community. It's one of the supervisory methods we normally use. (KCHW1)

However, the CHEWs who were interviewed felt that the reporting systems associated with referral needed to be strengthened, as they were unreliable.

There were occasions when the referral system did not work smoothly; for example, when a client was referred onwards by an health facility, it was not clear whose responsibility it was (the CHW's or the facility's) to ensure the client could reach the new facility.

There were also exceptional incidents in which CHWs had to act in ways that were demanded by the community, but for which they had not been trained. Some of these exceptions are relatively easily dealt with because of the informal networks developed by the CHWs, such as having a nurse's phone number.

There was an expectant woman who fell ill at night and in the morning I had to refer her. I called a nurse at the facility and told her about the mother. When I got at the facility I found out that she had been well received and attended to and she delivered. (MCHW2)

In some of these cases, nurses were able to attend rather than clients having to be sent to the health centre.

Mobile phones were critical in times of crisis, particularly when arranging transport for critically injured members of the community. However, this can place CHWs in a stressful position as the community may expect them to arrange transport to the health centre even when they are not present.

Sometimes there is fire, somebody has been burned. From here to that place is almost 300 meters and it was within my area. One of the 25 CHWs called me and told me somebody has burned come and help so I was far like 20 km away. So she asked me to go, mobilise people and I had resources in my phone. So I tried to handle the situation. I tried to send please call me but I couldn't manage because of no specific point of communication that were using and we are using even today. You are using your available resources for the purpose of helping that client who has had been burned. (KCHW1)

Further stress arises from discovering situations – some horrific – that fall outside their training, but which cannot be ignored, such as child abuse.

Sometimes you go to a forum and you are moved with the situation, you get a forum whereby mothers have been really beaten, children have been raped by their parents and you don't know how to handle it. Mine is on managerial level. I just want to go to the police and let the police take over. But the community there doesn't like that matter to be taken to the police instead they want to handle in the community. So there is a conflict. (KCHW2)

#### Educational interventions

Another important part of the work that CHWs described involved changing peoples’ environment and behaviour in ways that would improve their health.

It is my role to make sure all the people within my area are doing what we have learned all together. (KCHW1)

As part of their visits, the CHWs would advocate that people create and use latrines, make ‘leaky tins’ for washing, use malaria nets, have hospital births, use chemicals to treat water and so on.

I teach and help them construct dish racks and leaky tins, I show them the importance of mosquito nets so that they don't fall sick as they spend a lot of money in the hospitals. I also emphasis on hygiene and sanitation in the household and we make refuse pits and specific ones for disposal of sharps. (MCHW2)

Many of these interventions were seen as being successful and sustainable, because most of them only required materials that were ready to hand; those that relied on additional resources (such as the chemicals for water treatment or malaria nets) were harder to sustain.

When you listen to the community, you hear them talking about health hazards, leaky tins and chemicals for water treatment. So what the CHWs are doing is taken positively in the community. (MCHC)

Previously it was though that such things and acts like hand washing after visiting the toilet are a preserve of the well to do. Now when you go to latrines you don't have to ask for water because it is there. (MCHC)

Not all such interventions are directly health related; some CHWs also advocate other beneficial changes; for example, one CHW described having intervened in a row between a couple over sexual health, and others described environmental interventions.

On a daily basis as a CHW I go to my households which am allocated and I pass not only on health matters. For those mothers who have school going children I also advise them to take their children to school. Also as a CHW I see to it that my structures are clean so that the environment is comfortable for everyone. Sometimes maybe once or twice in a month I organise a cleanup, so we clean the area surrounding my structures with the community not alone. (KCHW3)

#### Monitoring communities

After working to change peoples’ behaviour or to refer them to a health facility, CHWs need to ensure that these changes persist. They regularly check on their clients to see whether they are continuing to act as recommended. Some of this is done through routine visits, for example, the follow-up checks with clients who have been referred. Other parts of this policing role are done by inspecting the households for evidence that the changes have been enacted.

So we normally link one another by dedicating specific themes to specific days, for example sanitation and hygiene particularly either by looking at the toilet, drainage systems, hand washing facilities within the area in the households. (KCHW1)

Such monitoring is closely linked to the data collection that CHWs undertake, in that the CHWs report to the CHCs on a regular basis, and submit data to the CHEWs. However, that work is reported separately, below. The monitoring most closely linked to data collection concerns disease surveillance, rather than the kinds of environmental change described above. It also involves regular checks on ongoing conditions, and reporting of exceptional incidents such as gender violence.

In the structure immediately you identify a pregnant mother […] you give her information about danger signs before delivery and after delivery […] you have to follow up to see if she has come across any danger signs and how she is handling it and if there is any problem. (KCHW2)

#### Data collection

The CHWs form a link between daily practice and strategic planning, ‘a link within our health centre, the facility and the community’ (KCHW1). Through their visits and monitoring, they generate data that are then submitted to others (primarily the CHCs and the CHEWs) to act on.

The data collection process is structured by Ministry of Health forms (MoH 513, 514 and 515, possibly transposed into a small booklet by Amref), which require information about who is in a household (a register, expected to be taken every 6 months), and also a wide range of situations including child health, maternal health, chronic conditions and disabilities, as well as infrastructure such as latrines, client activities such as breastfeeding or school attendance, CHW activities such as health talks and so on.

So when we enter in a household you start with the date, individual, name of the household, age. You ensure you start with the head of the household to the last born and you indicate everything in the household. (KCHW4)

I inquire from the household member available. There is a section for pregnant women, children under five years, for referrals I have made, check up for the elderly, whether the household has Information Education and Communication materials, whether the CHEW/CHC has visited the household, whether the household gets heath education through me, TB, and immunisation and vitamin A supplementation for the under fives, MUAC for assessing nutritional status of children. (MCHW2)

Additional information is sometimes required when special projects are running.

One CHW pointed out that completing such forms requires information from clients; they explained that such information is more freely given when the CHW visits the clients regularly, perhaps because they have developed a supportive and trusting relationship. There are also issues with the representativeness of the data set, which only covers established houses that have been allocated to a CHW.

One of our biggest challenges is that as you can see there are many buildings coming up and they are not registered. So it's a bit of a challenge to us because we don't get information from these households. (KCHW2)

The CHEWs receive these forms, usually once a month, and enter the data into the District Health Information System or the Community Based Health Management Information System so that the Ministry can access it. (One interviewee said that they went to the office to enter the data themselves.)

The data that are gathered then serve a dual purpose, monitoring both the community and the CHW and their interventions. For example, the standardised forms serve as prompts for the CHW, aligning their practice; the credibility of the data also prompts supervisors to check on the CHW's practice.

He sometimes visits or during submission of reports. They have to look at the reports as we are expected to be collecting information from the households and not sit under a tree and cook the information. (MCHW3)

The aggregated data are also used to assess, on a quarterly basis, whether the CHWs have had any impact on the area. They are also intended to form the basis of feedback from the supervisor to the CHW, although workloads mean that this is not always possible, and a more selective approach has to be adopted instead.

Actually to be honest you see you are talking of about 78 CHWs […] I want to see what 20 CHWs are doing then I look at that, then I give the books and I give the recommendations so that by the time we reach another meeting what I'll do is that I'll be able to note what somebody did which was good or which was not good like for example [A CHW] claimed that 13 clients out of 19 clients were having TB, so in short after having seen this report I will be able to give a feedback and when we have a meeting I will be able to give that feedback back because I don't think this is a true reflection of what happened. (KCHW3)

One CHW described that it also enabled them to remember what had been done on previous visits, and to check on their practice with their supervisors.

Whenever I go to a household am supposed to know what I have dealt on and what I've not. It's for my benefit and record and also whenever I give monthly reports I refer e.g. whenever there is a new born in my structures and that one is supposed to be reported during my monthly reporting so I refer to my black book the name of the kid when the kid was born. (KCHW3)

#### Resources

Many of the CHW's activities are circumscribed by resource issues, such as the lack of forms, medicine or free airtime for their mobile phones. The CHWs expected to be under-resourced.

We were also given a bag which has the CHW kits and of course it had a first aid kit that was not equipped. (KCHW2)

This was compounded by the fact that all the CHWs who were interviewed were volunteers, which meant that they had to rely on other sources of income to subsidise their work (for example when purchasing a mobile phone and airtime); and at the same time, they were giving up opportunities to earn income. Understandably, this situation gave rise to some emotional responses.

It is important for Amref to support us so that we do not feel like quitting. Because when I leave my house especially at a time like this when it's raining I should be in my farm tendering my crops but I haven't done that. So I ask Amref to give us whatever support they can to motivate us. Also, the Amref can provide us with first aid items so that we can provide first aid to our people before getting them to hospital. (MCHW2)

However, their clients did not seem to share this expectation, which caused them difficulties. In some cases, the clients demanded painkillers, or refused to believe the CHWs were volunteers, and insisted that they share their pay with them, levelling accusations that ‘we have eaten the money’ (KCHC). One CHC reported that ‘we have to carry flour or sugar for them’ as a way of buying their goodwill.

You can go into a household and they ask what it is that you have brought with you that time. If you have nothing they ask why you are there then. They complain that our job is just registering them all the time without any pay. (MCHW2)

They were provided with some equipment, although this was limited.

First of all we have been given black books so any case we get or anything we go to teach the community we note down and also we have referral forms for referring. We were given an umbrella in case of rain and also we were given gumboots. (KCHW3)

Moreover, some of these items were substitutions – for example the black books instead of Ministry forms. Other resources, such as the mobile phones, were provided by the CHWs themselves, and some resources they generated collectively – for example, creating reference notes after training sessions, where none had been provided. One CHEW also talked about writing proposals to attract funding for work, which they felt would give their community a degree of independence.

We will not be expecting Amref to be coming all the time because it's not only Amref that works on the ground. […] If they could sponsor me and do proposal writing, that would be a plus so that I would be able to sit with the communities we develop a proposal together, I'll be able to guide them and then they will be able to face those organisations as those community units and not as Amref. (KCHEW1)

Similarly, a CHW described how ‘If I had the ability to know more about) starting income generating activities it would be good for me to help [my community] as well to uplift their living standards’ (MCHW1). They also acted charitably towards each other where they could. One CHEW described how his colleagues had each given 100 shillings to create a fund that could be shared amongst the CHWs, and a CHC described how they organised a fund raiser to meet the costs of surgery for a CHW who had breast cancer.

The lack of equipment has direct consequences for health work, for example, preventing first aid, water treatment, diagnosis and referral.

If I get the MUAC tape, I will go and visit the child and assess. I place the tape on the upper arm and read. The reading needs to be in between. If it goes beyond or below normal then the child has a problem. [...] We will be given by Amref when they give us the kit. [So for now you do not have the MUAC?] No I don't. (MCHW2)

One CHW explained that having a torch would help, because it would enable them to work at night; another pointed to infrastructural problems, and explain how ‘there are no good drainages and in terms of doing cleanups we lack tools like rakes wheelbarrows so in case am provided with those ones it could help as a community as a whole’ (KCHW3).

Given the scarcity of resources, their control becomes an important issue. Access to phones or cameras provided by projects, for example, was a matter that one CHC wanted control of.

We use the CHC to keep those things and monitor them. So when you say about bringing mobile phone may be to the facility that will be hard work to us. You have to trust the CHC to keep even if it is one phone. If there is anything they want to report through the phone then they can come either to the secretary or chairperson but you can't say that it can't be entrusted to one person. Here in the slums it is very easy to lose an item, so you must entrust them to the CHC and they will know how to manage those items even if it is a camera. (KCHC)

The issue of trust is clearly important here, with the implication being that CHCs are more trustworthy than individual CHWs.

Transport was a recurrent issue. Mostly, the interviewees discussed walking, carrying patients on stretchers, improvising transport or sometimes paying fares; one CHEW mentioned an ambulance, but this was not considered an option by the CHWs. This limited referral work but also CHW's ability to visit remote households. One CHC suggested that a motorbike would make a real difference in enabling such visits.

We also monitor the stretchers. It's we as CHC who take care of them because when we are dealing with patients there we use stretches and ambulances; they are modern wheelbarrows with mattresses. We call them community ambulance. (KCHC)

Taken together, these resource issues complicate the work of the CHW immensely.

When we are referring, now internally there we have no roads. We [CB1]normally use manpower to take that client from the community up to here. It happens sometimes our client is referred from here to another district which is far and you want to go there and you have no cash, no modes of transport like motorcycles, no modes of communication, like maybe I have a phone which cannot communicate to the other side. Those items if they are available the job will be easier. (KCHW1)

#### Successes

A challenge for any policy-driven intervention is how to account for its success. In the interviews, CHWs spoke with pride about the work that they did for their communities, but it was rare to hear them speak in concrete, measurable terms about their successes and the impact they are having on the health of their community. Some of their successes are difficult to identify, because they involve changed practice, which may well pass unnoticed during evaluations.

The success is that they did not know the importance of hand washing after visiting the toilet, or from any work, or before preparing food. Now I am happy that I have taught them and they have leaky tins. Those who did not have functional toilets they build them. I am happy they are uplifting their health status. (MCHW4)

### Training and development of CHWs

#### The need for training

There were clear differences between CHWs about their experiences of training. Many viewed it very positively:

The trainings were good. […] Whatever we covered in the training has really helped me to do my work in the community as a CHW. (KCHW3)

For example, this supported their development as educators within their community, not only providing health messages for them to communicate, but also techniques to demonstrate, discuss and collaborate on, such as disease identification, maternal health talks and the construction of dish racks, leaky tins and so on.

The CHWs emphasised the importance of ongoing training, including refresher courses for familiar tasks. They were appreciative of the ongoing efforts to support training.

Since 1999 we have had so many trainings and so many certificates for CHW because we were trained on the basics of health as a whole. The CHWs who were trained in 1999, we were given basic skills for health as a whole, dispensing, home-based care and so much on health awareness. In the year 2003, we were trained as CHWs on HIV awareness, opportunistic infections and how to care for TB. From there we used to go for refresher courses not after a long period on home-based care, how to nurse a wound, dispensing but now it is not doing so well. (KCHW4)

However, a key stumbling block to the development of CHWs is that training is often haphazard, arising from projects run by different organisations.

We normally meet with some of officers from the government who may come, some of staffs from Amref; it depends on who is coming to roll out the event. It may be APHIAplus, Amref, NACADA, parastatals. (KCHW1)

This led to the view that projects use CHWs for their own needs – although this was tempered by the knowledge they their role remains key, if projects are to succeed with community engagement.

It also became clear that training over the last 3 years had become less frequent – indeed, some CHWs reported not having had any training in that period. Additionally, the training they did get was sometimes delivered in a manner that they could not keep up with.

I think am not well empowered. I need that knowledge because the training we were given the time frame was not adequate enough so it was like we are going fast to cover the topic and did not understand it properly. (KCHW2)

The CHWs combatted this through day-to-day support from CHEWs and each other, but they clearly desired more training to improve their practice.

It is when I visit households with people more educated than me. At times I may read and not fully understand while they understand better than me. At such a time I call my CHEW or CHW for assistance. When I appear not knowledgeable it's not so good, so if I get more training I will be better than I am this time. (MCHW2)

CHWs also reported that the quality of training varied depending on when it was taken and who was providing it. They were particularly concerned that training should address practical issues.

There is a gap in knowledge if you look at the CHWs who went for training in 2009 and 2003 on HIV infection, since the knowledge we had that time is not the same that was given to the CHWs who were recruited during the MNCH project. During the project we went for training in maternal and newborn. […] The training was intense and we even went for practicals […] and we were being assessed by nurses. (KCHW4)

A particular area of concern arose because the CHWs are volunteers, and not part of the formal health system. Historically, the CHWs had been attached to larger hospitals, but they were now associated with local clinics. From a health systems points of view this may be advisable, but for CHWs, it was also associated with a drop in training quality.

CHWs consistently expressed a need for more training. They felt a strong desire to be empowered, to have a better understanding of the health issues they encountered and to be able to connect this with the cultures of their communities. This would make a difference, for example, in their referral work.

[I]f we could be having more seminars on community health work, especially on diseases and their signs, then you will know as you take a child to hospital that they will be given this kind of medication. For now, I am no different from the ordinary community member who takes a child to hospital for treatment. But if I know that this disease needs this kind of medication then I will know if I have been given the right kind of medication for that disease. (MCHW2)

Finally, many wanted to receive training to work with technology.

I would like to update my computer literacy for further interactions of computer. When I get it, it will be good since I can save all information of CHW within the specific or internationally or locally. (KCHW1)

#### Supervision

The supervisory relationship was a particularly important feature in CHW's accounts, both in relation to assuring and enhancing the quality of their work.

Mostly, CHWs had a positive view of CHEWs’ role in supporting their practice. CHWs typically met their CHEW once a month to discuss problems (e.g. ‘If we meet an issue that we want to bring to the attention of the CHEW’, KCHW4), although some said that meetings were more common around specific projects. The commitment, support and expertise of CHEWs were valued.

They call us and if we have any challenges they help us resolve them. It has never occurred that our challenges go beyond the CHEW. If we can't handle the challenges, we call them and we resolve the issues together. (MCHW3)

However, some CHWs wanted CHEWs to shadow them in order to provide feedback on their practice.

Once in a while not always because for me most of the time I visit my households very late in the evening because they go for work so for the CHEW to come to my place, he does not stay around, so it's not very much possible for him to come and supervise when am working. Maybe I call him or he comes just once in a while. (KCHW3)

Others saw CHEWs as more managerial than educational, with feedback focused on reporting and data collection, rather than on caring for their communities:

The role of the CHEW is to provide direction. They also consolidate our reports and submit them. We ask questions as a community or when they check our reports and they notice a problem with them then they explain it to us as a group. (MCHW2)

#### Collective action between CHCs, CHEWs and CHWs, and the community

There is a close collaboration between CHCs, CHEWS and CHWs. The nature of this collaboration depends on the function of each person involved.

In all our meetings we are required to ask the CHEW to come. They are normally situated at the facility; Amref clinic and the district hospital are our facilities but our CHEW is situated here. Whenever we meet we invite him to come and if we have the monthly report he can take it then. (KCHC)

This close collaboration seems important not only in terms of division of labour, but because different roles bring different knowledge:

I think it is good to strengthen the CHEWs because they are our eyes in the facility and we are their eyes in the village. We could not have known if you are here if it were not for the CHEW. When there are new things in the facility, they do their work and they come and inform us. We communicate very well and we think it's good to strengthen them. (KCHC)

This sense of the collective goes beyond functional requirements for action and can create an environment of mutual support.

As the chairman I have one of my CHWs who has breast cancer and so I organised the CHWs, CHCs and CHEWs to a fund raiser to meet the cost of surgery. To make work continue even in her absence, I combined two villages to cover for her. (MCHC)

Indeed, interdependence can be seen throughout CHW's practice, for example when arranging transport for a referral.

We have a sick person in the community and we want to take him, he is bedridden, there are no roads there and we want to get that client to the facility. I will use my available resources to call you and I will mobilise the members in that specific area we take that client in that machela (stretcher) and we take that responsibility up to the health centre. (KCHW1)

It is also a reflection of the conditions and risks that all members of the community face, since everyone can be affected, directly or indirectly, by outbreaks of disease.

We also interact with them in the event of an outbreak. All the community members come together and we own it. Another reason why we interact with other community members is that if I am not infected am affected; if my family member dies because of an outbreak am also affected in one way or another. If we were working together as a CHW and one CHW dies, we are affected and we cannot continue working during that time of burial. So we need to interact with each other when anything comes out or comes in. (KCHW1)

#### Action days and dialogue days

Action days and dialogue days are important to strengthen collaboration and participation, within the community as well as between the volunteers.

[The action day] is a day when we come together all CHWs, CHC and CHEWs. We work together; we had one action day of sweeping the market. It was very successful and the people around commented that it was very clean. (MCHW4)

Dialogue days cover core areas of work – reporting, for example, or sharing issues that have arisen in practice.

The dialogue days this is where the whole community comes together and the chief is also involved plus the District Officer and we share with them the information we have been collecting. We put them on the wall so they just come and see and if they have any question they ask and then we tell them to come out with a major problem of which they want us to tackle. If they say it's a cleanup we do clean up or immunisation we do that. (KCHW2)

These collective meetings allow people to provide updates on changes and developments in their areas.

One of our biggest challenges is that as you can see there are many buildings coming up and they are not registered. So it's a bit of a challenge to us because we don't get information from these households. So that is the information we talk to our area leader. (KCHW2)

They also play an important role in terms of development – both of the volunteers and the community. Working together, CHWs are able to be more persuasive.

It is when I visit households with people more educated than me. At times I may read and not fully understand while they understand better than me. (MCHW2)

The advantage is that they usually listen to visitors more than the people from that village. So when I invite my neighbour they will pay attention and maybe they will accept. Even if someone doubted, they will accept and pay attention to what is being said. (MCHW4)

#### Other commitments of CHC, CHWs and CHEWs

The challenge for many CHWs is that, because their work is unpaid, they must maintain other sources of income.

Within my schedule of work, I am a teacher by profession, a hotelier and also an engineer; all those are my professions. What am doing in my community is that I have allocated specific time as a volunteer within my community. (KCHW1)

Many CHWs also have family commitments, which can lead to problems accessing others’ families at a convenient time.

During the morning hours I sometimes have a lot of work to do in my household before I leave. At time you are alone and all the kids have gone to school. I have to clean and leave everything in order before you leave. In most of my households the members work in the morning and come back in the evening. Sometimes I organise myself and go to the households and find no-one, forcing me to return in the evening. That is a challenge. (MCHW2)

These different demands intersect, making scheduling even more complicated.

Besides being a CHW, I am housewife, I do farming, I do the rest of the work in the house. I usually wake up very early in the morning and I take breakfast. I have some cattle so I get food for them. (MCHW4)

A further issue is that, although their work is unpaid, CHWs often feel obliged to buy equipment or supplies from their own pockets, as was noted earlier.

## Discussion

The study reported here supports contemporary policy commitments to the value of CHWs, demonstrating the work they do in integrating health and welfare initiatives in low-income, CTC settings, confirming the conclusions drawn in previous, initiative-specific studies [e.g. (1, 2)]. However, there was no evidence here of Lehmann & Sanders’ differentiation (5) between generalists and specialists; all CHWs involved here could be seen as generalists, who acted as specialists in response to specific funded programmes, but whose role extended beyond this, providing continuity for the community whilst funding comes and goes.

What this analysis confirms, consistently, is that the CHWs play a crucial role in the success of community health initiatives. This point is well established [e.g. (5)]; however, this study develops this point in two ways. Firstly, reflecting calls to recognise the expertise of CHWs (4), this study has shown that CHWs are able to provide detailed insights into their work, and secondly, their accounts have served to identify specific ways in which success was achieved in spite of the well-documented challenges of geography, resourcing, training and so on. Through community support, relationships with medical practitioners, personal efforts and sacrifices, the CHWs coped, improvised and worked together to serve their communities. The relational aspects of CHWs’ roles have long been recognised as important [e.g. (6)], but the details of what these relationships consist of, how they operate and how they enable success has not been closely described.

The integrative role of CHWs is particularly important within their accounts. As Ofose-Amaah proposed (8), CHWs do link communities and health facilities; as this study has shown, they achieve this through referrals and the provision of data, but also in more material ways, such as by arranging transportation. They also raise issues and challenges that can then be taken up by CHEWs and CHCs, who are better positioned to lobby for funding or resources. Their role is also key to the success of external initiatives, as they come to embody links between funded programmes and community life.

However, this linking role required negotiating tensions between obligations to the formal healthcare system and to communities. Tensions were identified, for example, between the ‘policing’ function implied in their data collection and monitoring work, and the trust needed for clients to share sensitive or troubling issues, which might be understood as part of a ‘social work’ role. Such tensions may be inherent in such ‘linking’ roles, which will have implications for future interventions or developments; these tensions are not recognised in existing literature, even when both roles are identified [e.g. (13)]. However, some related issues have been identified. For example, Lehman & Sanders (5) note the ongoing tensions between understanding community participation as the *mobilisation* of a community's resources (people, money, materials) and the *control* they can exercise over the social, political, economic and environmental factors determining their health. However, they do not discuss the ongoing enactment of this tension in CHWs’ day-to-day work. Similarly, Mireku et al. (13) describe the mistrust that can arise when communities suspect CHWs are withholding resources.

Various authors have raised questions about CHW's training. This study confirmed that CHWs see this as vital, as something that distinguishes them from being just another concerned member of the community, but felt that training has been inconsistent and of variable quality. As Kahssay et al. suggest (6), training can be provided through supervision, formal courses, team-based approaches and increased community involvement. Whilst formal courses may be lacking, there is evidence that CHWs are addressing this issue themselves by pursuing community-led possibilities such as action and discussion days. Supervision is present, but appears to be inconsistent, and more detailed feedback on practice was requested.

In line with other empirical studies, such as those by Sharma et al. (7) and Nyamhanga et al. (14), these interviews draw attention to the material culture of CHW's work. Being a CHW is not simply a matter of role or training, but also an issue of bicycles, leaky tins, stretchers, water purification tablets, mobile phones and so on. The scarcity of resources shapes practices and informs priorities; however, it also creates ways of attributing success and exercising power through controlling access. The way in which a CHC wanted to keep control over phones or cameras provided by a project illustrates this; consolidation of responsibility of access to such devices confirmed their position of power relative to the CHWs.

This is not simply a matter of giving CHWs more – although more medical resources would clearly be welcomed – but also of understanding the way that scarcity is linked to such questions of access, and also to sharing, collective action (e.g. when carrying a stretcher to a health centre) and a sense of community. Many examples were provided of the kind of mobilisation that Lehman & Sanders (5) identify; however, whilst they remain cautious about expecting CHWs to take responsibility for mobilising communities, the accounts here suggest that this happens regularly, albeit on an *ad hoc* and modest basis.

Although resources are always likely to be limited in low-income settings, a more developed appreciation of cultures and practices would allow better informed decisions to be made around which resources to prioritise, and how they should be distributed. This is particularly clear in the Kenyan case, where there is a commitment to resourcing CHWs at a policy level but, as this study has shown, this does not always happen in practice. This paper shows the nature of this divide and how CTC providers work to overcome it. This deeper understanding is not only important for outlining the nature of the serious divide between policy and practice but is key to developing ways in which to address it.

## Conclusions

Although the use of CHWs is an important response in low-income settings to shortfalls in health provision, the ways in which they enact this role has been relatively poorly described and understood. By studying the day-to-day practices of CHWs in two regions of Kenya, and documenting their accounts and explanations of these practices, this paper has helped address this gap, providing rich accounts of what CHWs do; who they do it with; and what they need in order to do it. In doing so, it has helped address the call (4) for the expertise and insight of CHWs to be taken into account in policy and health interventions. The depth and detail of the themes presented here constitute an addition to existing work in the field, which has not provided a similarly ‘thick description’ of practices.

CHWs do enact the priorities of externally funded health initiatives within communities, reaching out to the community, beyond formal, funded institutional environments. This is, however, only a small part of their work. The greater part entails attending to and caring for households within their community, by diagnosing them (in relatively constrained ways), referring them, monitoring them, educating them, in some cases supplying them, and by representing them to others who have greater strategic or economic reach. In so doing, they bind health provision to communities, crossing repeatedly between formal health systems and communities’ day-to-day existence. Their work does give them some status within the community, but they remain marginal within health provision, seem insecure about their status (partly because they feel they lack specialist medical expertise) and may need to turn to existing community structures of power and authority to shore up their position when their work is challenged. They are actively supported by figures of authority within their community and in the health service – although these individuals can also judge and criticize their performance and manner.

This work is carried out in spite of many material and organisational challenges, including geography, and the shortage of training and resources. The CHWs’ strategies show resourcefulness, creativity and persistence; rather than being a problem in need of ‘fixing’, characterised in terms of deficits, CHWs’ own accounts show them as active agents in achieving the success of health initiatives – albeit agents operating within difficult material circumstances, and facing complex relationships of power, for example in relation to who gets to judge their work or controls access to resources. Overcoming the challenges of implementation required CHWs to bring together community members, elders and health specialists in order to advise, carry, enforce, educate and so on. They also have to manage their responsibilities as CHWs alongside other commitments, such as farming, other jobs or their own family life, since the unpaid status of their work requires them to maintain other forms of support.

Recognising the complexity of their role – including its social and logistical elements, as well as its medical ones – and respecting the expertise required to cope with implementation challenges is important for future attempts to support or develop CHWs. Interventions that ignore the social credibility they require, or which misinterpret the links between community authority and the control of resources, risk failing, or even undermining their ongoing work.
